# The Frequency of Epidermal Growth Factor Receptor (EGFR) Mutation in Patients with Lung Adenocarcinoma Referred to a Lung Diseases Hospital; A Cross-Sectional Study from Iran

**DOI:** 10.30699/IJP.2022.533427.2673

**Published:** 2022-02-20

**Authors:** Sotoudeh Mohammadi, Mitra Rezaei, Fatemeh Shojaeian, Mihan Pourabdollah, Leila Mohammadi Ziazi, Sharareh Seifi, Atousa Doroudinia, Babak Salimi, Adnan Khosravi, Mohammad Amin Farhangnasab

**Affiliations:** 1 School of Medicine, Shahid Beheshti University of Medical Sciences, Tehran, Iran; 2 Department of Pathology, School of Medicine, Shahid Beheshti University of Medical Sciences, Tehran, Iran; 3 Chronic Respiratory Diseases Research Center, National Research Institute of Tuberculosis and Lung Disease (NRITLD), Shahid Beheshti University of Medical Sciences, Tehran, Iran; 4 Department of Pathology, National Research Institute of Tuberculosis and Lung Disease (NRITLD), Shahid Beheshti University of Medical Sciences, Tehran, Iran; 5 Clinical Tuberculosis and Epidemiology Research Center, National Research Institute of Tuberculosis and Lung Disease (NRITLD), Shahid Beheshti University of Medical Sciences, Tehran, Iran; 6 Tobacco Prevention and Control Research Center, NRITLD, Shahid Beheshti University of Medical Sciences, Tehran, Iran

**Keywords:** Adenocarcinoma, EGFR Mutation, Frequency, Iranian population, Lung cancer, Smoking

## Abstract

**Background & Objective:**

Various studies showed the use of epidermal growth factor receptors (EGFRs) gene mutations in the therapeutic plan of patients with advanced lung cancer. This study aimed to investigate the frequency and types of *EGFR* gene mutations among Iranian patients with lung adenocarcinoma referred to a specialized lung diseases hospital from 2014 to 2019.

**Methods:**

The data of all patients with lung adenocarcinoma referred to the Molecular Department of Masih Daneshvari Hospital Laboratory (National Research Institute of Tuberculosis and Lung Diseases) from 2014 to 2019 for *EGFR* mutation tests were collected. Patients' characteristics data and information on the frequency and types of *EGFR* gene mutations were obtained from the hospital information system (HIS). The collected data were analyzed using SPSS 25.

**Results:**

A total of 570 individuals (Mean age of 58.74, 51.6% Male) were included in the study; 113 out of 570 patients (19.8%) were diagnosed with gene mutation. In terms of the type of mutation, 65 participants (57%) showed deletion, 48 patients (42.1%) were diagnosed with replacement, and one (0.9%) case demonstrated both. Notably, the mutation rate detected among the female patients was significantly higher than the male ones (*P*=0.001); in particular, deletion type of mutation was found more among women, although both genders were the same in terms of the replacement frequency. However, the age had no effect on the mutation in this study (*P*=0.05).

**Conclusion:**

Among Iranian patients with lung adenocarcinoma, 19.8% harbored EGFR gene mutation. This mutation was found in association with lung cancer and could affect the patient's therapeutic plan.

## Introduction

Lung cancer is one of the frequent causes of cancer-related death,. In 2012, a total of 1.6 million indivisuals in the world died of lung cancer and the number of worldwide deaths is predicted to rise to 3 million by 2035 (1). It showed that the total number of breast, prostate, and intestinal cancer victims is lower than that of patients who died from lung cancer (2). However, limited health care resources in developing countries make a barrier for observation the disease's accurate prevalence and mortality rate. Lung cancer has four major histopathological types divided into small-cell and non-small-cell lung cancer, accounting for about eighty percent of pathologic types (3,4).

As the most frequent malignant neoplasm in most countries among both sexes, lung cancer has some advances in different types of treatment, such as chemotherapy, radiotherapy, and surgical approaches. Still, the long-term survival rate remains low (5), with an overall survival rate of 1% and a 5-year survival rate of 3.5% (6). However, some critical risk factors, including tobacco consumption, lung fibrosis, genetic susceptibili-ty, poor diet, air pollution, and occupational exposure, seem to be correlated with future lung cancer involve-ment (7–9).

Advances in studying biomarkers as a quantitative factor in diagnosing and treating many diseases result in personalized medicine and appropriate therapeutic plans and drugs for each patient (10–12). One gene that affects lung cancer is the Epidermal Growth Factor Receptor (*EGFR*) (13). It is a membrane protein with tyrosine kinase activity with various functions, such as cell growth, proliferation, and differentiation (14). Studies have shown the correlation between *EGFR* mutation and different cancers, which activate the cell surface receptor (15–17). Besides, this protein would increase cell survival by inhibiting apoptotic pathways (18,19). Consequently, it has been shown that the *EGFR* muta-tion is correlated with the non-small-cell lung cancer patients' response to therapy, such as Erlotinib and Gefitinib, as *EGFR* inhibitors (20–22). 

Previous studies have confirmed the impact of this mutation on deciding the appropriate therapeutic plan for the patients. Moreover, it has been stated that the frequency of mutation of *EGFR* is different among the different populations, and it might be related to the race; for instance, a study has shown that the mutation frequency is 2% and 26% among American and Japan-ese population, respectively (18–21,23). Hence, the appropriate response to *EGFR* inhibitors is supposed to differ among people. 

In Iran, lung cancer is the second and third cause of cancer-related death in men and women, respectively, and the country is struggling with the disease (24,25). However, there is a lack of investigation about the EGFR mutation among Iranian patients with lung cancer, influencing the selected therapeutic plan. A study in 2018 revealed the frequency of 24.3% EGFR mutation among 103 lung cancer patients (24). In the current study, 570 patients with lung adenocarcinoma referred to the National Research Institute of Tuberculosis and Lung Diseases (NRITLD) were investigated for EGFR mutation to identify the frequency of this mutation among Iranian lung cancer patients.

## Material and Methods

The current research was a retrospective descriptive study. The data of patients with lung adenocarcinoma referred to the Masih Daneshvari hospital, Tehran, Iran (National Research Institute of Tuberculosis and Lung Diseases (NRITLD)), from 2014 until 2019, were gathered. Patients' demographic characteristics (inclu-ding age, sex, and city), history of the disease, and therapeutic plan were obtained from Hospital Inform-ation System (HIS). The patients were referred to the Pathology and Molecular Department for genomic investigation.

These patients had undergone a biopsy, or the specimens were resected surgically. The mutation analysis was conducted on formalin-fixed paraffin-embedded (FFPE) tissues samples from primary or metastatic sites. The genomic DNA was extracted, and exons 18, 19, and 21 of the EGFR gene were amplified using polymerase chain reaction (PCR). Analysis of the genomes was performed in line with company protocols. These molecular analysis data were avail-able at the hospital information system (HIS).

The investigation was performed following the Declaration of Helsinki's ethical standards and national and international guidelines, approved by the Institu-tional Review Board of Shahid Beheshti University of Medical Sciences. Written informed consent was also obtained from all the patients. 

The data are shown as mean ± standard deviation (SD). Statistical analysis was conducted using the chi-square test and the student's t-test, comparing categorical variables and independent groups. A P-value <0.05 was considered statistically significant. All analyzes were performed using SPSS 25.0 (IBM, Armonk, NY, USA)**.**

## Results

**Demographic Data**

**Fig. 1:**
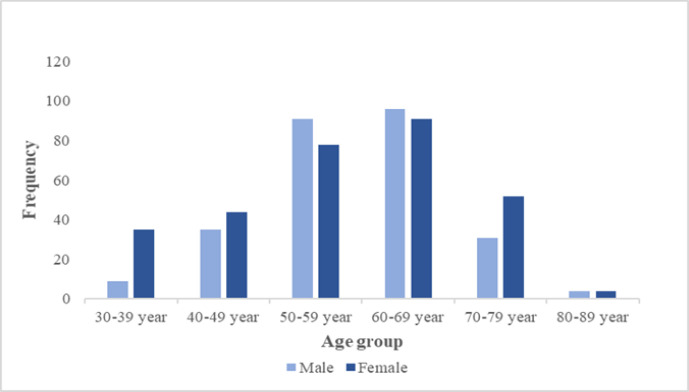
The frequency of age groups (among male and female)

A total of 570 participants were referred to the molecular and pathology department with the lung cancer diagnosis and included in this study (Table 1). Altogether, 58.74±11.84 was the mean age of the patients. In terms of gender, men and women consti-tuted 294 (51.6%) and 276 (48.4%) of the participants (Figure 1). The mean age of the men was 59.8 ± 10.48, and the mean age of the women was 57.81±12.92. Hence, the patients' mean age was not notably different between male and female candidates (*P*>0.05).

**The Frequency of EGFR Mutation Among Participants**

Among 570 participants, 113 patients (19.8%) had *EGFR* mutation; 57% of the mutations were in the form of deletion, 42.1% were in the state of replacement, and 0.9% of mutations, had both replacement and deletion. Most *EGFR* mutation was detected in exon 19 (54.4%), followed by exon 21 (36.8%). Moreover, 7% of the mutations were detected in exon 18, and 1.8% were in two exons. Table 2 depicts the frequency of mutation in different exons.

**Table 1:** Patients' demographic characteristics

Subject	Variables
Number of Patients	**570**
Male	**294**
Female	**276**
Age (average)	**58.74 ± 11.84**
Former or Current Smoker	**312 (54.7%)**
Never Smoker	**258 (45.3%)**

**Influence of Gender on Mutations**

Among all 113 detected mutations, men and women were constituted 43 and 70 of the mutations, respectively. As shown in Table 2, the mutation rate was significantly higher among female candidates than males (*P*=0.001). In detail, the frequency of exon 19 mutation was significantly higher in women (*P*=0.02) than in men (Table 3). Moreover, among all deleted nucleotides detected (65 cases), 27.7% and 72.3% were found in men and women. Hence, the men's and women's difference in deletion frequency was significant (*P*=0.016). Conversely, the difference between men and women in terms of replacement mutation was not significant (*P*>0.05), which was detected in 22 men (45.8%) and 26 women (54.2%).

**Table 2:** The frequency of each type of mutations in different exons and genders

	Types of Mutation		
	**Deletion**	**Replacement**	**Deletion and Replacement**
18	0	8	-
19	61	1	-
21	4	38	-
18 & 19	0	0	1
21 & 19	0	1	0
Male	18 (27.7%)	26 (54.2%)	0
Female	47 (72.3%)	22 (45.8%)	1 (100%)
Total	65	48	1

**Table 3:** The frequency of each exon mutation among men and women

	Sex		
	Male	Female	**P-value**
**18**	5 (62.5%)	3 (37.5%)	**>0.05**
**19**	15 (24.2%)	47 (75.8%)	**0.02**
**21**	23 (54.8%)	19 (45.2%)	**>0.05**
**19 & 18**	0	1 (100%)	**-**
**19&21**	**1 (100%)**	**0**	**-**

**Relation of Mutation with Smoking and Age**

A total of 312 (54.7%) were smokers among all the participants, and 258 (45.3%) patients were non-smokers. The *EGFR* mutation rates were 31% and 69% in smokers and non-smokers group, respectively (odds ratio [OR], 0.29 [95% CI, 0.18–0.45]; *P*<0.0001). So, the *EGFR* mutation showed a notable difference between smokers and non-smokers candidates.

Various age groups had no significant differences in the rate of deletion and replacement (*P*>0.05). Besides, the types of mutated exons were not significantly different in these age groups (*P*>0.05).

## Discussion

The current study assessed the frequency and types of mutations in epidermal growth factor receptor (*EGFR*) genes in patients with lung adenocarcinoma. Following this evaluation, *EGFR* gene mutations were observed in 19.8% of all participants. Most of these mutations (57%) were nucleotide deletions, and the replacement was the following category (42.1%). In one case (0.9%), nucleotide deletion and replacement were observed simultaneously.

Mutations are not limited to a change in just one nucleotide (replacing one nucleotide with another); they also include deletions, insertions, and duplication, which necessitates the genome study in different diseases, including cancer. Among all types of neoplasms, lung cancer is the most frequent cause of cancer-related death in Americans. Also, the number of deaths due to this fatal cancer is higher than the total number of deaths due to breast, prostate, and colorectal cancer together (2). Hence, it is essential to investigate lung cancer, looking for possible mutations affecting the diagnosis, treatment, and prognosis, as the deadliest cancer globally. 

One of the biological changes that might be associated with lung cancer is the mutation of the EGFR oncogene (25). Epidermal growth factor receptor (EGFR) is a protein of the cell membrane, which has tyrosine kinase activity, with various functions, such as cell growth, proliferation, and differentiation (21). Numerous studies showed a relation between the *EGFR* gene polymorphism and advanced stages of various cancers, such as lung and gastric cancer (26, 27). The presence of a mutation in the *EGFR* gene activates the receptor on the cell surface (17). Besides, this receptor induces cell survival by inhibiting apoptotic pathways (18, 19). *EGFR* mutation is revealed to be related to response to the receptor antagonist drugs (such as gefitinib) in patients with non-small cell lung carcinoma (NSCLC) (26, 27). Studies have also shown that allelic forms of this gene are involved in lung cancer (28, 29). In addition, the gene polymorphisms of this receptor might be related to race and geographical conditions and vary in different populations, so the rate of polymorphism in the American people is 2% and in the Japanese population is 26% (18,20). Therefore, it seems essential for each country and region to investigate the rate and frequency of this mutation among people.

In the current study, in 113 out of 570 samples (19.8%), EGFR mutations have been detected. However, in Iranian patients diagnosed with esophageal cancer, the incidence of EGFR mutation was higher, in such a way that Lashkarizadeh* et al.* reported 82% mutations among all 60 samples (30). In our study, 65 cases (57%) of all mutations were in the form of deletion. In 48 patients (42.1%), nucleotide replacement was present, and a combined nucleotide deletion and replacement coexisting were observed in only one case (0.9%). In the study of Lashkarizadeh* et al.*, in 52% of cases, the mutation was of the gene deletion type; in 30% of the cases, the mutation was seen as gene duplication, and in other cases, both types of mutation were detected simultaneously (30). In this study, most of the deletion occurred in exon 19 (about 94%), while most of the replacement occurred in exon 21 (about 79%). In the study of *EGFR* mutations in esophageal cancer patients, most of the deletion mutations were in exon 2 (44%), and the highest rate of replacement mutations (54%) was in exon 27 (30). In terms of smoking, most of the mutated cases were among non-smoker participants (69%), and the difference in mutation among smokers and non-smokers was significant. The results were in line with the previous studies, which showed a higher mutation rate in non-smoker patients (31,32).

In a similar study by Basi *et al. *on lung adenocarcinoma, *EGFR* mutations were observed in 25 out of 103 patients (24.3%), which is inconsistent with the value obtained in our study (24). In Basi *et al.*'s study, the most common sites of mutations were exon 21 (15 patients; 60%) followed by exon 19 (10 patients; 40%); although in our study, it was the other way around, and the mutations in exon 19 (62 patients; 54.9%) occurred more frequently than exon 21 (42 patients; 37.2%), following by exon 18 mutation (8 patients; 7.1%). In this study, the overall mutation incidence in women (70 patients; 61.9%) was significantly higher than in men (43 patients; 38.1%). It was in contrast with the study of Basi* et al.*, in which the incidence of mutations was equally distributed between men and women (24). In this study, the rate of deletion was significantly higher in women. Still, there was no significant difference between these two genders regarding replacement mutations. However, in the study of Basi *et al.*, no similar investigation has been done. 

Investigating blood, urine, and tissue biomarkers are becoming appropriate for early cancer detection (33,34). Accordingly, various researches were performed on the possibility of using tumor biomarkers for lung cancer screening. Plasma microRNAs, circulating tumor cells, and autoantibodies are presented as possible biomarkers for lung cancer diagnosis (35–37). In other studies, carcinoembryonic antigen (CEA) serum level in non-small-cell lung cancer patients was higher than the other types of cancer (38,39). Moreover, another survey of 184 patients revealed a relation between lung cancer involvement and CK19 and CEA serum levels (40). Hence, advances in biomarkers and genomic fields are considered the future of cancer investigation, and *EGFR* as an important proven marker could be used in this regard. Consequently, its frequency in different populations could play an essential role in planning treatment guidelines for lung cancer in each country. 

In conclusion, the association of the *EGFR* gene mutation with lung cancer has been indicated, so investigation of *EGFR* mutation could help decide about patients' therapeutic plan, such as anti-*EGFR* drug usage. The frequency of *EGFR* mutation in lung adenocarcinoma patients referred to a specialized lung disease hospital has been investigated. A higher frequency among Iranian patients than the western population was obtained, although the frequency seems almost the same as the eastern population.

Further multi-centric studies with a higher number of participants, other genomic evaluations, and investigating the possible application of this mutation for screening and early detection of cancer among the different populations are recommended. 

## Conclusion

The frequency of *EGFR* mutation among the Iranian population with lung adenocarcinoma referred to a specialized lung disease hospital is 19.8%, and it is higher among female patients than males. Most of the mutations were deletion and presented in exon 19.

## Conflict of Interest

The authors declared no conflicts of interest.

## Funding

None.
